# Moderate-intensity stepping in older adults: insights from treadmill walking and daily living

**DOI:** 10.1186/s12966-023-01429-x

**Published:** 2023-03-18

**Authors:** T. Yates, J Henson, P. McBride, B Maylor, L. Y. Herring, J. A. Sargeant, M. J. Davies, P. C. Dempsey, A. V. Rowlands, C. L. Edwardson

**Affiliations:** 1 Diabetes Research Centre, College of Life Sciences, University of Leicester, Leicester, UK; 2 NIHR Leicester Biomedical Research Centre, University Hospitals of Leicester NHS Trust and University of Leicester, Leicester, UK; 3 Leicester Diabetes Centre, University Hospitals of Leicester National Health Service Trust, Leicester, UK

**Keywords:** Cadence, Metabolic equivalents (METS), Older adults, Physical activity, Resting metabolic rate, Stepping

## Abstract

**Background:**

A step cadence of 100 steps/minute is widely used to define moderate-intensity walking. However, the generalizability of this threshold to different populations needs further research. We investigate moderate-intensity step cadence values during treadmill walking and daily living in older adults.

**Methods:**

Older adults (≥ 60 years) were recruited from urban community venues. Data collection included 7 days of physical activity measured by an activPAL3™ thigh worn device, followed by a laboratory visit involving a 60-min assessment of resting metabolic rate, then a treadmill assessment with expired gas measured using a breath-by-breath analyser and steps measured by an activPAL3™. Treadmill stages were undertaken in a random order and lasted 5 min each at speeds of 1, 2, 3, 4 and 5 km/h. Metabolic equivalent values were determined for each stage as standardised values (METS_standard_) and as multiples of resting metabolic rate (METS_relative_). A value of 3 METS_standard_ defined moderate-intensity stepping. Segmented generalised estimating equations modelled the association between step cadence and MET values.

**Results:**

The study included 53 participants (median age = 75, years, BMI = 28.0 kg/m^2^, 45.3% women). At 2 km/h, the median METS_standard_ and METS_relative_ values were above 3 with a median cadence of 81.00 (IQR 72.00, 88.67) steps/minute. The predicted cadence at 3 METS_standard_ was 70.3 (95% CI 61.4, 75.8) steps/minute. During free-living, participants undertook median (IQR) of 6988 (5933, 9211) steps/day, of which 2554 (1297, 4456) steps/day were undertaken in continuous stepping bouts lasting ≥ 1 min. For bouted daily steps, 96.4% (90.7%, 98.9%) were undertaken at ≥ 70 steps/minute.

**Conclusion:**

A threshold as low as 70 steps/minute may be reflective of moderate-intensity stepping in older adults, with the vast majority of all bouted free-living stepping occurring above this threshold.

**Supplementary Information:**

The online version contains supplementary material available at 10.1186/s12966-023-01429-x.

Brisk walking is a powerful predictor of all-cause mortality and cardiovascular disease [1–5], with recent data also showing associations with a lower risk of severe COVID-19 [6]. Research to date has predominately utilised self-reported walking pace or functional walking tests. However, the widespread use of accelerometers within research and commercial activity trackers has enabled measures of walking pace applicable to daily living. Specifically, step cadence has been used as a measure of walking intensity with a threshold of 100 steps/minute used to identify moderate-intensity stepping [7], including in associations with health outcomes [8–11]. The recent CADENCE study, which directly counted steps during treadmill walking with oxygen consumption measured by indirect calorimetry, went on to systematically confirm that 100 steps/minute acts as an evidence-based heuristic threshold in all adults [12, 13], including older adults [14].

Generalisability of the 100 steps/minute threshold needs confirming in older adults, particularly as it has been shown that the metabolic cost of walking is higher than in younger populations, with low walking speeds of < 3 km/h achieving > 3 metabolic equivalents (METS) [15–17]. This supports a hypothesis that step cadence thresholds for moderate-intensity stepping may be lower in older adults. The CADENCE study did not support this hypothesis as the walking pace required to achieve 3 METS was consistent to that reported for the general population (~ 4 km/h) [14, 18], which may explain why the moderate-intensity step cadence threshold of 100 steps/minute was also consistent with younger populations [12, 13]. Therefore, it is important to further investigate the generalisability of the 100 steps/minute threshold to a wider population of older adults.

The aim of this study was to investigate moderate-intensity step cadence values during treadmill walking in older adults and to use data collected during daily living to quantify stepping cadence behaviour in relation to derived thresholds.

## Methodology

### STAND-UP study

The analysis included data from the ‘Sedentary behaviour in older adults: investigating a new therapeutic paradigm (STAND UP) study’ [19]. One of the work packages consisted of a cross-sectional study collecting accelerometer data during free-living conditions followed by a lab-based assessment of different physical activities. The aim was to develop age-appropriate cut-points for sedentary behaviour and physical activity definitions in older adults. Participants were recruited from the community by working with a network of social clubs, faith centres and other community venues from 2014 to 2016. Participants were eligible to take part if they were ≥ 60 years of age, able to walk without assistance from support devices or other persons, and able to communicate in verbal and written English. Those with self-reported chronic disease likely to affect participation in or outcomes of the study were excluded (e.g. asthma, diabetes, heart disease, memory problems).

### Visits and measures

#### Visit 1

The first visit was organised to collect baseline data, confirm eligibility, and undertake familiarisation with the study procedures including treadmill walking. Data collection included height and weight (to the nearest 0.5 cm and 0.1 kg respectively) and a 60-s sit-to-stand test of physical function, where participants were instructed to stand from a standardised sitting position as many times as possible within 60 s. At the end of this visit participants were fitted with an activPAL3™ device (PAL Technologies, Ltd., Glasgow, UK) on the midline anterior aspect of the right thigh and asked to wear the device continuously for 7 full days. Devices were waterproofed using a nitrile sleeve and affixed to the thigh using Hypafix Transparent (BSN Medical, Hull, UK) dressing.

#### Visit 2

Following collection of baseline data, participants attended an exercise and metabolic laboratory (Diabetes Research Centre, Leicester General Hospital, United Kingdom) in a fasted condition. Participants first completed a 60-min resting metabolic rate (RMR) protocol whilst supine. Expired gas was collected from a calibrated open circuit breath-by-breath Cortex Metalyzer (Leipzig, Germany) using the low flow rate setting. The initial 30 min acclimatised participant to the laboratory and equipment and the final 30 min was used for the REE measurement. The first 5-min period where the average per minute VO2 and VCO2 changed by less than 10% was used to calculate REE. Following REE measurement, participants consumed a standardised breakfast (men 500 kcal, women 400 kcal) and rested for a further 30 min. Participants then undertook a series of activities typical of daily living. This included treadmill walking at 1, 2, 3, 4, and 5 km/h, with each stage lasting 5 min, performed in a randomised order. During each phase of the test, breath-by-breath expired gas was collected with data between minutes 2 to 4 averaged for analysis (Cortex Metalyzer, Leipzig, Germany). An activPAL3™ device was worn throughout.

### Device-assessed physical activity and sedentary behaviour

activPAL3™ devices were initialised using default settings (Professional Research Edition; PAL Technologies Ltd., Glasgow, UK). For the free-living data, event files were processed using Processing PAL (Version, 1.3, University of Leicester, UK), which uses a validated algorithm to determine the waking wear time within each valid day [20]. A valid waking day was defined as a day with < 95% of time spent in any one behaviour (e.g., standing or sitting), ≥ 500 step events (1000 steps/day) and ≥ 10 h of valid waking hours data [20]. At least 3 valid days of data were included for the free-living assessment. Cadence (steps/minute) was calculated per step event as ‘(number of steps × 2/interval length) × 60’. Cadence for continuous uninterrupted bouts of stepping, as a marker of purposeful walking, were calculated as bouts lasting a minute or longer and calculated as ‘(number of steps × 2/interval length) × 60’. Bouted steps above different step cadence thresholds anticipated to cover the threshold for moderate-intensity within this population (50–100 steps/minute in 10 unit increments) were extracted for analysis. For the laboratory visit, 1 s epoch files were created from the event files in order to extract stepping data for minutes 2 to 4 of each walking stage to match the METS data. ActivPAL devices have previously been shown to accurately count steps during treadmill and continuous walking within controlled conditions, including at slower speeds in older adults, with an absolute percentage error of < 1% [21, 22], with another study reporting that over 90% of steps above 69 steps/minute are counted [23].

### Metabolic equivalent (METS) values

METS were calculated using data from minutes 2 to 4 of each treadmill stage. METS_standard_ were calculated using the standardized formula: VO_2_ (mL/kg/min)/3.5. METS_relative_ were calculated by dividing VO_2_ (mL/kg/min) during each treadmill stage by the resting metabolic rate VO_2_; unlike METS_standard_, METS_relative_ accounts for individual differences in VO_2_ during rest and thus provides a multiple of resting energy expenditure. A value of 3 METS_standard_ defined the threshold for moderate-intensity stepping.

### Data inclusion

Sixty-two individuals completed the study, of which 58 undertook at least one stage of the treadmill test, with 4 individuals excluded for safety reasons related to balance and proprioception. activPAL malfunction or difficulties with monitor placement on frail skin excluded a further 5 participants, with 53 providing valid activPAL data included in the analysis.

### Statistical analysis

Segmented generalised estimating equations modelled the association between step cadence and MET values, taking into account repeated measures using an exchangable correlation matrix. A breakpoint at 100 steps/min was selected as it provided the best model fit, consistent with the previous literature [14]. As the focus of this paper is on the threshold for moderate-intensity stepping, the regression line up to 100 steps/minute was used for this analysis (see Supplementary Figure S1). Equations were used to predict step cadence at 3 MET_standard_ and 3 MET_relative_ along with METS_standard_ and MET_relative_ values at 100 steps/minute. In order to assess whether results were consistent across different characteristics, models were stratified by sex (women, men), age (categorised at the median), BMI (< 30, ≥ 30 kg/m^2^), height (categorised at the sex stratified median), physical function status (categorised at the median) and physical activity status (< 7500 steps/day, ≥ 7500 steps/day) for all outcomes apart from step cadence at 3 METS_relative_ which were not investigated further due to predicted values falling outside the range of plausible purposeful stepping (< 50 steps/minute). Receiver Operating Characteristic (ROC) curve analyses were performed to further test the strength of association between step cadence and moderate-intensity classification. Sensitivity, specificity, positive predictive values (PPV) and negative predictive values (NPV) were generated for the derived step cadence threshold for moderate-intensity and compared to those observed for a threshold of 100 steps/minute; values were corrected to account for repeated measures using generalised estimating equations [24].

Data are presented as median (IQR) for descriptive data or estimate (95% CI) for modelled data. Data were analysed using IBM SPSS Statistics (version 24.0). A *p*-value of < 0.05 was considered statistically significant.

## Results

The study included 53 participants (median age = 75, years, BMI = 28.0 kg/m^2^, 45.3% women), with characteristics displayed in Table 1.

**Table 1:** Participant characteristics

**Categorical characteristics**	**Number**	**Column %**	
Sex			
Men	29	54.7	
Women	24	45.3	
Ethnicity			
White European	45	84.9%	
Other	8	15.1%	
**Continuous characteristics**	**Median**	**25**^**th**^ **percentile**	**75**^**th**^ **percentile**
Age (years)	75	68	78
Body Mass Index (kg/m^2^)	28.0	26.6	29.9
Lower limb function (sit-to-stand repetitions)	19	16	23
Resting VO_2_ (ml/kg/min)	2.77	2.52	3.02
Total ambulatory activity (steps/day)	6988	5933	9211
Ambulatory activity at ≥ 1 min bouts (steps/day)	2554	1297	4456

### Lab data

MET and step cadence value for each stage of the treadmill test are displayed in Table 2. At 2 km/h, the median (IQR) METS_standard_ and METS_relative_ values were 3.08 (2.73, 3.43) and 3.62 (3.27, 4.30) respectively at a cadence of 81.0 (72.0, 88.7) steps/min.

**Table 2:** Metabolic equivalent and stepping cadence values during treadmill walking

Treadmill speed	METS_standard_			METS_relative_			Cadence (steps/minute)		
	Median	25^th^ Percentile	75^th^ percentile	Median	25^th^ Percentile	75^th^ percentile	Median	25^th^ Percentile	75^th^ percentile
1 km/h	2.29	2.12	3.07	2.88	2.47	3.53	64.7	49.3	85.3
2 km/h	3.08	2.73	3.43	3.62	3.27	4.30	81.0	72.0	88.7
3 km/h	3.55	3.15	4.24	4.47	3.93	5.02	94.7	86.7	99.3
4 km/h	4.15	3.69	4.85	5.28	4.56	5.85	108.7	100.7	114.0
5 km/h	4.52	4.08	5.32	5.56	5.10	6.43	115.3	105.3	123.3

The predicted METS_standard_ and METS_relative_ value at 100 steps/minute, based on 200 data observations across the treadmill stages, were 3.77 (95% CI 3.54, 4.01) and 4.74 (4.43, 5.04) respectively. With a gradient of 0.026 METS_standard_ and 0.032 METS_relative_ per 1 step/minute difference in cadence, the predicted cadence value at 3 METS_standard_ or METS_relative_ were 70.3 (61.4, 75.8) steps/minute and 45.8 (27.7, 56.6) steps/minute, respectively (Supplementary Table S1, Supplementary Figure S1). Values were largely consistent across age, sex, BMI, height, physical function and physical activity status (Supplementary Table S1).

The ROC curve for the association between MET classification and stepping cadence is shown in Supplementary Figure S2, with an AUC value of 0.814. A threshold of 70 steps/minute correctly classified (true positive and true negative values) 80% of all observations, with a high sensitivity and PPV (93.4% and 82.6%, respectively) (Supplementary Table S2). However, the NPV (64.4%) and specificity (37.6%) were lower. Conversely, a threshold of 100 steps/minute had lower accuracy (62.4% of all observations correctly classified), low sensitivity (54.6%), but reasonable specificity (87.1%) (Supplementary Table S2).

### Free-living data

**Fig. 1:**
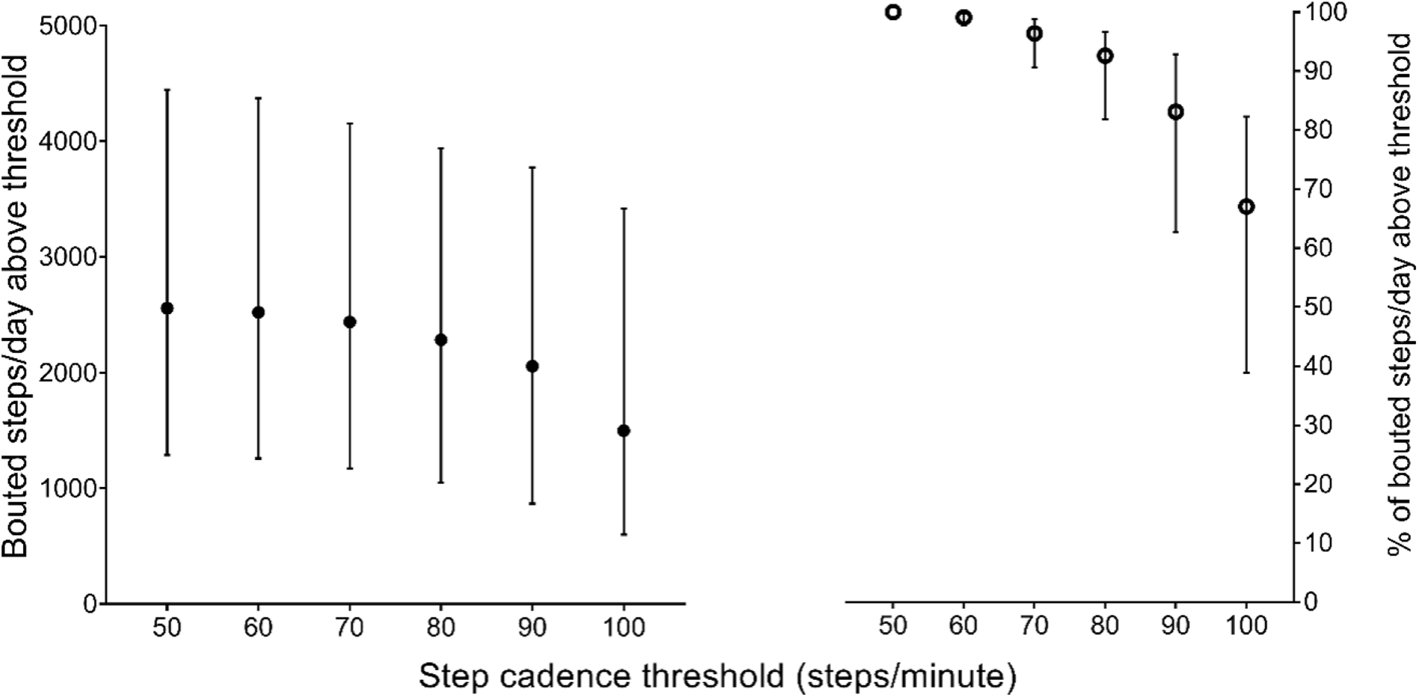
Number and percentage of bouted steps taken at or above incremental cadence thresholds during daily living. Data as median (IQR)

Participants undertook a median (IQR) of 6988 (5933, 9211) steps/day, of which 2554 (1297, 4456) steps/day were undertaken in continuous stepping bouts of 1 min or more (Table 1). Figure 1 shows the amount and proportion bouted daily steps undertaken at or above incremental thresholds from 50 to 100 steps/minute. In total, 96.4% (90.7, 98.9) of all bouted steps were undertaken at or above a cadence of 70 steps/minute, with 67.0% (38.9%, 82.3%) undertaken at or above 100 steps/minute.

## Discussion

In this analysis of older adults, the threshold for moderate-intensity physical activity was reached at a stepping cadence of 70 steps/minute which was substantially below the widely used 100 steps/minute, with the lower threshold providing higher levels of accuracy and sensitivity, but lower levels of specificity. These findings have application to free-living conditions as the vast majority of bouted stepping during daily living occurred above 70 steps/minute.

Our threshold is lower than that reported in the CADENCE study [12–14], which concluded that 100 steps/minute acts as a heuristic threshold for all adults, including older adults aged 60–85 years [14]. However, the prediction interval for the CADENCE study did include 70 steps/minute, suggesting some overlap in the range of predicted threshold between studies. There are some differences in population characteristics that may help explain the difference the mean predicted thresholds. Most notably, the walking speed needed to elicit METS_standard_ varied substantially between our studies. In the CADENCE study, average values above 3 METS_standard_ were only seen at walking speeds of around 4 km/h. However in our study, 3 METS_standard_ was exceeded at half this speed (2 km/h). Therefore whilst the gradient in the association between step cadence and METS_standard_ was similar between studies (0.026 vs 0.020), the difference in walking speed needed to elicit 3 METS_standard_ resulted in a substantially different intercept and step cadence thresholds. Previous literature supports the findings in our cohort, with slow walking speeds consistently shown to result in elevated METS_standard_ values in older adults [15–17, 25, 26], and slow walking speeds of < 3 km/h shown to cross the threshold into moderate-intensity activity [15–17]. The reasons for the difference in the metabolic cost of walking with age are not fully understood, although age-related adaptations in the recruitment and activation of leg muscles and excess body weight have been proposed [27, 28]. Indeed, median BMI values in our cohort were higher than the CADENCE study (28.0 vs 26.7 kg/m^2^) and more reflective of older adults within the general population [29].

The importance of defining the step cadence threshold for moderate-intensity physical activity in older adults is highlighted by the inclusion of free-living purposeful (bouted) stepping behaviour within this study. Over 90% of bouted steps occurred above 70 steps/minute. Therefore, if the threshold for moderate-intensity stepping exists at around 70 steps/minute in older adults, a focus on increasing continuous stepping activity is likely to a priori also promote moderate-intensity physical activity. This has important implications for public health campaigns and physical activity promotion interventions and tools where a focus on simply promoting increased walking time or distance may be optimal. This is supported by observational studies showing that associations between step cadence and health outcomes are largely attenuated after adjustment for overall stepping volume [9, 11], including in older adults [8, 10]. However, if the threshold sits at closer to 100 step/minute, only around two thirds of continuous daily stepping is undertaken at a moderate-intensity, which may support a focus on targeting both the volume and intensity of walking for optimal health.

Within this older population, the RMR VO_2_ was 2.77 ml/kg/minute, which is consistent with other studies and substantially below the value used to calculate METS_standard_ (3.5 ml/kg/minute) [30], which was derived in younger populations. Consequently, METS_relative_ values for walking were higher than those for METS_standard_ with virtually all treadmill walking occurring above 3 METS_relative_, further supporting the conclusion that any continuous stepping occurs at a moderate-intensity in older adults.

Our analysis has some strengths and limitations. Strengths include the use of activPAL devices to collect both free-living and treadmill walking activity, allowing for interpretation and application of generated cadence thresholds into routine daily activity. However, accelerometer devices in general may not capture all activity during light or infrequent stepping [23], and the use of activPAL may also present some limitations. Previous research, including in older adults, has shown good validity between observed and activPAL counted steps during treadmill or controlled slow walking (within the range used in this study [21–23]), providing reassurance that misclassification of steps is unlikely to account for the magnitude of difference between our study and the CADENCE study, which amounted to approximately 30 steps/minute. Moreover, a walking pace of 2 km/h elicited over 3 METs within this study compared to a walking pace of 4 km/h within the CADENCE study, therefore our findings are also consistent with a lower stepping cadence simply reflecting the slower pace required to achieve the moderate-intensity threshold. However, it is also possible that conducting treadmill walking in the postprandial state may have acted to elevate derived MET values, thus lowering the step cadence needed to achieve 3 METS. We did not capture metabolic data during free-living conditions, and are therefore unable to verify whether MET values are consistent between treadmill and free-living stepping at derived cadence thresholds. The relatively small sample size may not be generalizable to all older adults, or those with greater mobility limitations.

In conclusion, this study suggests that a threshold as low as 70 steps/minute may on average be reflective of moderate-intensity walking in older adults, with the vast majority of bouted (≥ 1 min) stepping occurring above this threshold. However, the low specificity at a threshold of 70 steps/minute highlights a higher risk of misclassifying low-intensity steps as moderate-intensity steps compared to 100 steps/minute, suggesting important trade-offs in the selection of the threshold used whilst also highlighting the need for further research to refine this threshold for moderate-intensity stepping in older adults and within other populations associated with accelerated aging, such as those living with long-term conditions.

## Supplementary Information

**Additional file 1: Supplementary Table S1.** Predicted values across participant characteristics. **Supplementary Table S2.** Levels of agreement between predicted stepping intensity and actual stepping intensity across 200 observations for a METS_standard_ definition of moderate-intensity. **Supplementary Figure S1.** Distribution of data points and regression lines. **Supplementary Figure 2.** Receiver Operating Characteristic (ROC) curve characteristics.

## Data Availability

The datasets during and/or analysed during the current study available from the corresponding author on reasonable request.

## Abbreviations

METS: Metabolic equivalents
METS_standard_: Calculated using the standardized METS formula: VO_2_ (mL/kg/min)/3.5
METS_relative_: Calculated by dividing VO_2_ during each treadmill stage by the resting metabolic rate VO_2_
REE: Resting energy expenditure

## References

[1] Yates T, Zaccardi F, Dhalwani NN, Davies MJ, Bakrania K, Celis-Morales CA, Gill JM, Franks PW, Khunti K (2017). Association of walking pace and handgrip strength with all-cause, cardiovascular, and cancer mortality: a UK Biobank observational study. Eur Heart J.

[2] Argyridou S, Zaccardi F, Davies MJ, Khunti K, Yates T (2020). Walking pace improves all-cause and cardiovascular mortality risk prediction: A UK Biobank prognostic study. Eur J Prev Cardiol.

[3] Liu B, Hu X, Zhang Q, Fan Y, Li J, Zou R, Zhang M, Wang X, Wang J (2016). Usual walking speed and all-cause mortality risk in older people: a systematic review and meta-analysis. Gait Posture.

[4] Tanasescu M, Leitzmann MF, Rimm EB, Willett WC, Stampfer MJ, Hu FB (2002). Exercise type and intensity in relation to coronary heart disease in men. JAMA.

[5] Manson JE, Hu FB, Rich-Edwards JW, Colditz GA, Stampfer MJ, Willett WC, Speizer FE, Hennekens CH (1999). A prospective study of walking as compared with vigorous exercise in the prevention of coronary heart disease in women. N Engl J Med.

[6] Yates T, Razieh C, Zaccardi F, Rowlands AV, Seidu S, Davies MJ, Khunti K (2021). Obesity, walking pace and risk of severe COVID-19 and mortality: analysis of UK Biobank. Int J Obes.

[7] Tudor-Locke C, Han H, Aguiar EJ, Barreira TV, Schuna JM, Kang M, Rowe DA (2018). How fast is fast enough? Walking cadence (steps/min) as a practical estimate of intensity in adults: a narrative review. Br J Sports Med.

[8] Brown JC, Harhay MO, Harhay MN (2014). Walking cadence and mortality among community-dwelling older adults. J Gen Intern Med.

[9] Saint-Maurice PF, Troiano RP, Bassett DR, Graubard BI, Carlson SA, Shiroma EJ, Fulton JE, Matthews CE (2020). Association of daily step count and step intensity with mortality among US adults. JAMA.

[10] Lee IM, Shiroma EJ, Kamada M, Bassett DR, Matthews CE, Buring JE (2019). Association of step volume and intensity with all-cause mortality in older women. JAMA Intern Med.

[11] Paluch AE, Bajpai S, Bassett DR, Carnethon MR, Ekelund U, Evenson KR, Galuska DA, Jefferis BJ, Kraus WE, Lee IM, Matthews CE (2022). Daily steps and all-cause mortality: a meta-analysis of 15 international cohorts. Lancet Public Health.

[12] Tudor-Locke C, Aguiar EJ, Han H, Ducharme SW, Schuna JM, Barreira TV, Moore CC, Busa MA, Lim J, Sirard JR, Chipkin SR (2019). Walking cadence (steps/min) and intensity in 21–40 year olds: CADENCE-adults. Int J Behav Nutr Phys Act.

[13] Tudor-Locke C, Ducharme SW, Aguiar EJ, Schuna JM, Barreira TV, Moore CC, Chase CJ, Gould ZR, Amalbert-Birriel MA, Mora-Gonzalez J, Chipkin SR (2020). Walking cadence (steps/min) and intensity in 41 to 60-year-old adults: the CADENCE-adults study. Int J Behav Nutr Phys Act.

[14] Tudor-Locke C, Mora-Gonzalez J, Ducharme SW, Aguiar EJ, Schuna JM, Barreira TV, Moore CC, Chase CJ, Gould ZR, Amalbert-Birriel MA, Chipkin SR (2021). Walking cadence (steps/min) and intensity in 61–85-year-old adults: the CADENCE-Adults study. Int J Behav Nutr Phys Act.

[15] Hall KS, Howe CA, Rana SR, Martin CL, Morey MC (2013). METs and accelerometry of walking in older adults: standard versus measured energy cost. Med Sci Sports Exerc.

[16] Barnett A, van den Hoek D, Barnett D, Cerin E (2016). Measuring moderate-intensity walking in older adults using the ActiGraph accelerometer. BMC Geriatr.

[17] Duncan MJ, Rowlands A, Lawson C, Leddington Wright S, Hill M, Morris M, Eyre E, Tallis J (2020). Using accelerometry to classify physical activity intensity in older adults: What is the optimal wear-site?. Eur J Sport Sci.

[18] Ainsworth BE, Haskell WL, Herrmann SD, Meckes N, Bassett DR, Tudor-Locke C, Greer JL, Vezina J, Whitt-Glover MC, Leon AS (2011). Compendium of physical activities: a second update of codes and MET values. Med Sci Sports Exerc.

[19] Yates T, Edwardson CL, Celis-Morales C, Biddle SJ, Bodicoat D, Davies MJ, Esliger D, Henson J, Kazi A, Khunti K, Sattar N (2020). Metabolic effects of breaking prolonged sitting with standing or light walking in older South Asians and white Europeans: a randomized acute study. J Gerontol A Biol Sci Med Sci.

[20] Winkler EA, Bodicoat DH, Healy GN, Bakrania K, Yates T, Owen N, Dunstan DW, Edwardson CL (2016). Identifying adults’ valid waking wear time by automated estimation in activPAL data collected with a 24 h wear protocol. Physiol Meas.

[21] Grant PM, Dall PM, Mitchell SL, Granat MH (2008). Activity-monitor accuracy in measuring step number and cadence in community-dwelling older adults. J Aging Phys Act.

[22] Hergenroeder AL, Barone Gibbs B, Kotlarczyk MP, Kowalsky RJ, Perera S, Brach JS (2018). Accuracy of objective physical activity monitors in measuring steps in older adults. Gerontol Geriatr Med.

[23] Stansfield B, Hajarnis M, Sudarshan R (2015). Characteristics of very slow stepping in healthy adults and validity of the activPAL3™ activity monitor in detecting these steps. Med Eng Phys.

[24] Genders TS, Spronk S, Stijnen T, Steyerberg EW, Lesaffre E, Hunink MM (2012). Methods for calculating sensitivity and specificity of clustered data: a tutorial. Radiology.

[25] Ndahimana D, Kim YJ, Wang CS, Kim EK (2021). Energy cost of walking in older adults: accuracy of the ActiGraph accelerometer predictive equations. Nurs Res Pract.

[26] Hooker SP, Feeney A, Hutto B, Pfeiffer KA, McIver K, Heil DP, Vena JE, LaMonte MJ, Blair SN (2011). Validation of the actical activity monitor in middle-aged and older adults. J Phys Act Health.

[27] LaRoche DP, Marques NR, Shumila HN, Logan CR, St Laurent R, Gonçalves M (2015). Excess body weight and gait influence energy cost of walking in older adults. Med Sci Sports Exerc.

[28] Hortobágyi T, Finch A, Solnik S, Rider P, DeVita P (2011). Association between muscle activation and metabolic cost of walking in young and old adults. J Gerontol A Biol Sci Med Sci.

[29] Health Survey for England 2018. Overweight and obesity in adults and children http://healthsurvey.hscic.gov.uk/support-guidance/public-health/health-survey-for-england-2018/overweight-and-obesity-in-adults-and-children.aspx. Accessed 27 Jan 2022.

[30] Hall KS, Howe CA, Rana SR, Martin CL, Morey MC (2013). METs and accelerometry of walking in older adults: standard versus measured energy cost. Med Sci Sports Exerc.

