# Supplementary material for: Moderate-intensity stepping in older adults: insights from treadmill walking and daily living
Source: Int J Behav Nutr Phys Act. 2023 Mar 18;20:31. doi: 10.1186/s12966-023-01429-x (PMC10024004; doi:10.1186/s12966-023-01429-x)
Supplement: Supplementary file 1 — Additional file 1: Supplementary Table S1. Predicted values across participant characteristics. Supplementary Table S2. Levels of agreement between predicted stepping intensity and actual stepping intensity across 200 observations for a METSstandard definition of moderate-intensity. Supplementary Figure S1. Distribution of data points and regression lines. Supplementary Figure 2. Receiver Operating Characteristic (ROC) curve characteristics. [file 12966_2023_1429_MOESM1_ESM.docx]

**Supplementary Table S1: Predicted values across participant characteristics**

|  | Predicted step cadence at 3 METS_standard_ | | | Predicted METS_standard_ at 100 steps/minute | | | Predicted METS_relative_ at 100 steps/minute | | |
| --- | --- | --- | --- | --- | --- | --- | --- | --- | --- |
|  | Value | Lower CI | Upper CI | Value | Lower CI | Upper CI | Value | Lower CI | Upper CI |
| **All** | 70.3 | 61.4 | 75.8 | 3.77 | 3.54 | 4.01 | 4.74 | 4.43 | 5.04 |
| **Sex** |  |  |  |  |  |  |  |  |  |
| Women | 74.8 | 63.6 | 80.7 | 3.66 | 3.32 | 4.00 | 4.77 | 4.26 | 5.28 |
| Men | 66.2 | 48.3 | 75.6 | 3.88 | 3.55 | 4.21 | 4.74 | 4.36 | 5.11 |
| **Age** |  |  |  |  |  |  |  |  |  |
| <75 years | 70.1 | 62.7 | 75.1 | 3.90 | 3.56 | 4.23 | 4.67 | 4.18 | 5.16 |
| ≥75 years | 67.7 | 12.3 | 80.2 | 3.61 | 3.29 | 3.94 | 4.76 | 4.39 | 5.13 |
| **Obesity** |  |  |  |  |  |  |  |  |  |
| No | 70.3 | 57.2 | 77.3 | 3.77 | 3.48 | 4.06 | 4.67 | 4.33 | 5.02 |
| Yes | 69.7 | 53.6 | 77.5 | 3.79 | 3.40 | 4.18 | 4.95 | 4.32 | 5.58 |
| **Height*** |  |  |  |  |  |  |  |  |  |
| Short | 67.8 | 46.3 | 77.6 | 3.81 | 3.50 | 4.11 | 4.59 | 4.18 | 5.00 |
| Tall | 72.6 | 63.1 | 77.6 | 3.74 | 3.39 | 4.09 | 4.87 | 4.43 | 5.32 |
| **Physical function** |  |  |  |  |  |  |  |  |  |
| Good | 70.5 | 60.2 | 75.8 | 3.80 | 3.51 | 4.09 | 4.78 | 4.36 | 5.20 |
| Poor | 69.9 | 19.8 | 81.0 | 3.72 | 3.31 | 4.13 | 4.65 | 4.19 | 5.11 |
| **Physical activity** |  |  |  |  |  |  |  |  |  |
| Active | 68.5 | 49.7 | 77.1 | 3.76 | 3.44 | 4.08 | 4.52 | 4.15 | 4.90 |
| Inactive | 72.2 | 61.1 | 78.9 | 3.78 | 3.46 | 4.10 | 4.86 | 4.45 | 5.27 |

* = Derived at the sex stratified median value (women = 157 cm, men = 170 cm)

**Supplementary Table S2: Levels of agreement between predicted stepping intensity and actual stepping intensity across 200 observations** **for a METS_standard_ definition of moderate-intensity**

|  | **Threshold used to define moderate-intensity stepping** | | | |  |
| --- | --- | --- | --- | --- | --- |
|  | **70 steps /minute** | | **100 steps/minute** | |  |
| **Actual MET value** | METS < 3 (N) | METS ≥ 3 (N) | METS < 3 (N) | METS ≥ 3 (N) |  |
| METS < 3 (N) | 18 | 30 | 43 | 5 |  |
| METS ≥ 3 (N) | 10 | 142 | 68 | 84 |  |
| **Accuracy, sensitivity, specificity, and predictive values** | | | | | |
|  | Standard | Corrected* | Standard | Corrected* |  |
| Accuracy (%) | 80.0 | 80.0 | 63.5 | 62.4 |  |
| Sensitivity (%) | 93.4 | 93.4 | 55.3 | 54.6 |  |
| Positive predictive value (PPV, %) | 82.6 | 82.6 | 94.4 | 93.1 |  |
| Specificity (%) | 37.5 | 37.6 | 89.6 | 87.1 |  |
| Negative predictive value (NPV, %) | 64.3 | 64.4 | 38.7 | 37.7 |  |

* = Corrected for repeated measures

**Supplementary Figure S1: Distribution of data points and regression lines**

**
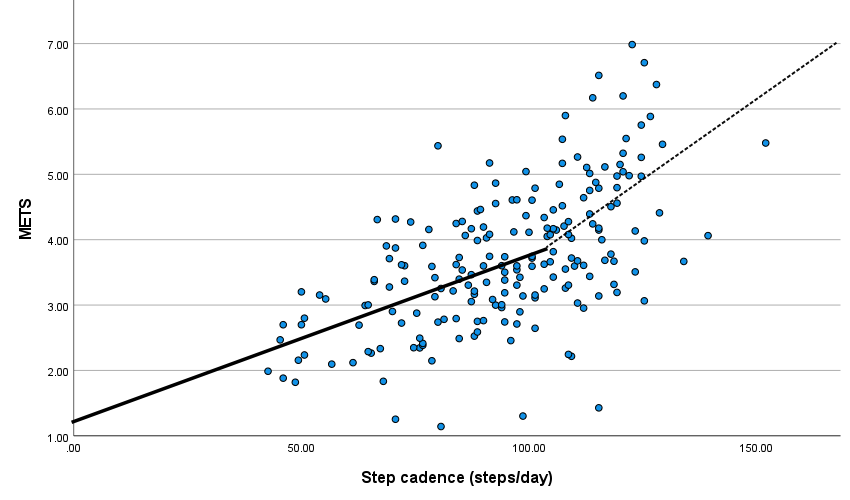
**

METS

Steps/minute

Bold line shows regression line up to the break point at 100 steps/minute, which was used to derive the data in this analysis. The regression line takes account of repeated measures.

**Supplementary Figure 2: Receiver Operating Characteristic (ROC) curve characteristics**


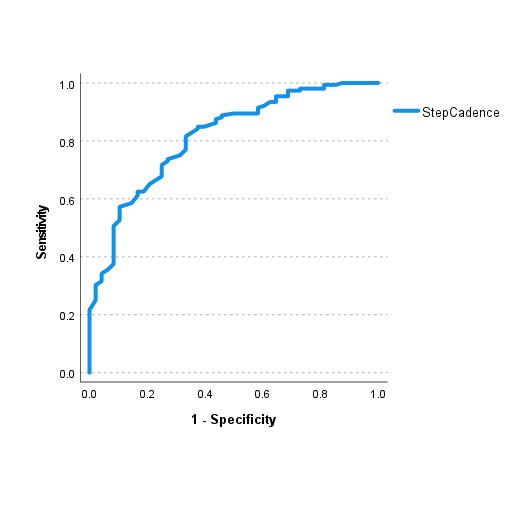


Area under the curve (AUC) = 0.81
